# Abnormalities in iNKT cells are associated with impaired ability of monocytes to produce IL‐10 and suppress T‐cell proliferation in sarcoidosis

**DOI:** 10.1002/eji.201344284

**Published:** 2014-05-12

**Authors:** Anjali Crawshaw, Yvonne R. Kendrick, Andrew J. McMichael, Ling‐Pei Ho

**Affiliations:** ^1^ Weatherall Institute of Molecular MedicineUniversity of OxfordOxfordUK; ^2^ Oxford Centre for Respiratory MedicineChurchill HospitalOxfordUK

**Keywords:** IL‐10, Invariant natural killer T (iNKT) cells, Monocytes, Sarcoidosis, T‐cell proliferation

## Abstract

Sarcoidosis is a multisystem granulomatous disorder characterized by marked T‐cell expansion of T helper 1 (Th1) cells. The cause of T‐cell overactivity is unknown. We hypothesized that interleukin‐10 (IL‐10) production by a yet undefined cell type might be defective, resulting in loss of regulation of T‐cell activity. Focusing on IL‐10‐producing monocytes, we first showed that monocytes isolated from the peripheral blood of corticosteroid‐naïve sarcoidosis patients (*n* = 51) produced less IL‐10 compared to controls, and were less able to suppress T‐cell proliferation. In addition, monocytic IL‐10 production correlated negatively with disease activity score. As invariant natural killer T (iNKT) cells are known to both interact with monocytes and be reduced in sarcoidosis patients, we then asked whether iNKT‐specific defects might be responsible for this reduced IL‐10 production. We found that greater numbers of circulating iNKT cells was associated with higher IL‐10 production. Moreover, iNKT cells enhanced monocytic IL‐10 production in vitro. Defective IL‐10 production and T‐cell suppression by sarcoidosis monocytes could be restored following their coculture with iNKT cells, in a CD1d‐ and cell contact‐dependent process. We suggest that reduced iNKT‐cell numbers in sarcoidosis may lead to impaired monocytic IL‐10 production and unchecked T‐cell expansion in sarcoidosis. These findings provide fresh insight into the mechanism of sarcoidosis disease, and interaction between iNKT cells and monocytes.

## Introduction

Sarcoidosis is a multisystem inflammatory condition characterized by granuloma formation. The lungs are affected in 90% of cases and are likely to be the first site of exposure to antigen 1. In the early phase of disease, T‐cell overactivity is marked and characterized by IFN‐γ, TNF‐α, and IL‐2‐producing CD4^+^ TH1 2. There appears clear compartmentalization in immune response in the blood and lungs — the former characterized by lymphopenia and relative anergy, while the T cells infiltrating the airways are highly activated with increased levels of IFN‐γ, TNF‐α and IL‐2 production. A role for exogenous antigens is suggested by reports that patients have an expansion of T cells with biased TCR usage in the lungs 3, 4, and findings of excess representation of pathogenic antigens (e.g. from *Mycobacterium* and *Propionibacterium* spp.) in sarcoid lesions 5, 6, 7, 8. These antigens are likely to act as triggers for the host's predisposition to inappropriate, large T‐cell responses. The cause of this aberrant T‐cell response is unknown but it is likely to be critical to the generation and maintenance of granuloma. Granulomagenesis first requires an intracellular antigen that is poorly degradable which is engulfed by macrophages 9 which then become ‘fusion‐competent’ 10. In some cases, (e.g. formation of multinucleated giant cells around mycobacterial epitopes) this process appears to be IFN‐γ dependent 10. Perpetuation of granuloma then requires several factors, a key factor being TNF‐α 11. Therefore, initiation and then maintenance of granuloma requires appropriate T‐cell ‘‘help’’ in the form of IFN‐γ and TNF‐α provision. Arguably, the exaggerated T‐cell response in sarcoidosis is the pivotal process in disease genesis, yet the cause of this uncontrolled T‐cell activity is unknown.

Genome‐wide association study had identified BTNL‐2, a butyrophilin/B7‐like molecule, a purported negative costimulatory molecule for T‐cell proliferation, as a potential susceptibility factor 12 but the function of BTNL‐2 in sarcoidosis is poorly understood and little functional work has been done to support this hypothesis. Another possible cause for the large CD4^+^ T‐cell expansion is a defect in IL‐10 producing cells, since IL‐10 has distinct T‐cell suppressive effect 13. These could be IL‐10 producing FoxP3 regulatory T (Treg) cells, regulatory B (Breg) cells 14 or the less studied, IL‐10 producing monocytes 15. Both Treg cells and Breg cells have been investigated in sarcoidosis but paradoxically shown to be elevated in numbers 16, 17. Very little is known of ‘‘regulatory monocytes.’’ IL‐10 producing monocytes were first reported in the mid 1990s’ when it was shown that these cells have self‐regulatory properties as the IL‐10 moderated autosecretion of IL‐1, IL‐6, IL‐8, and TNF‐α 15. Several papers subsequently established its existence 18, 19 but its role in host defence and immunopathology has never been clear. There is evidence that it is increased in atopic patients, respiratory syncytial virus infection, malignancy, and a recent paper suggests that IL‐10 produced by monocytes during HIV‐1 virus infection prevented T‐cell activation 20, 21. We are particularly interested in these cells because they are precursors to activated macrophages and granulomagenesis, and they are found in the vicinity of proliferating T cells 22. In addition, we, and others 23, 24, 25 have described abnormally low invariant natural killer T (iNKT) cells in sarcoidosis (and other T‐cell mediated diseases), and these cells are known to modulate monocyte function and influence outcome of T cell‐mediated diseases 26, 27. In a model of severe lung injury caused by influenza A virus infection, levels of the monocyte chemoattractant, MCP‐1 and inflammatory monocytes were markedly elevated in the lungs of iNKT knockout mice (Jα18^−/−^)^26^, while in experimental autoimmune encephalomyelitis (EAE), a model of multiple sclerosis, activation of iNKT cells deviated the differentiation of monocyte to noninflammatory/M2 macrophage with improvement in outcome 27. iNKT cells are a specialized subset of T cells that recognize self and foreign lipids presented by CD1d. They have both protective and harmful roles, depending on the pathological context, but are vital for optimal immune response to microbial infection and cancer. In sarcoidosis, the consequence of its deficiency is not fully understood. One suggestion is that they are involved in control of T‐cell proliferation but the mechanism has not been elucidated. Here, we ask if iNKT cells can control T‐cell activity via its interaction with monocytes. We first homed in on aspects of monocytes which may affect T‐cell function. We reasoned that since IL‐10 has powerful T‐cell suppressive activity 13, and monocytes can secrete IL‐10, abnormality in IL‐10 production could affect T‐cell activity. Findings in the first part of this study suggest this may be the case, and prompted further experiments to explore if reduction in iNKT cells impacted on IL‐10 production by monocytes.

## Results

### Monocytes from patients with sarcoidosis have reduced capacity to produce IL‐10

We first isolated monocytes from blood of 51 patients who were not on any treatment at the point sampling (and for at least 3 months before). All patients had pulmonary sarcoidosis and were nonsmokers. Of the 51 patients, two were African‐Caribbean, three South Asian (India/Pakistan) and the rest were Caucasians. All had pulmonary involvement, one had lung, heart, and uveitis, five had lung and skin, 12 had lung and uveitis, two had lung and cardiac sarcoidosis. Infectious agents were excluded in all patients and specifically, cultures of lung lavage fluid were negative for mycobacterium tuberculosis.

Because we and others have found that monocytes were activated using Ficoll separation 23, whole blood was first red cell‐lysed, and subjected to isolation of ‘‘untouched’ monocytes using CD14 MACS^TM^ bead negative selection kit. Samples were used immediately after venepuncture to provide the best level of functional preservation.

**Figure 1:**
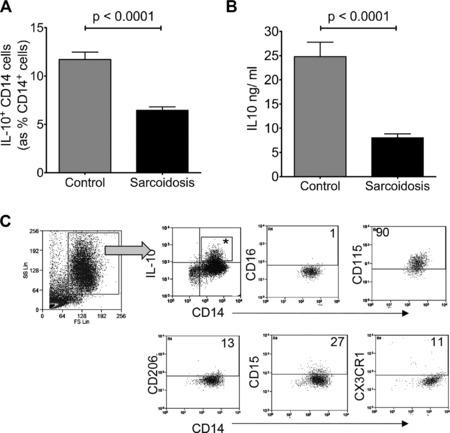
Monocytes from patients with sarcoidosis produce less IL‐10. Freshly isolated monocytes from patients with sarcoidosis and from healthy donors were stimulated with lipopolysaccharide (LPS) and analyzed for interleukin‐10 (IL‐10) expression by (A) intracellular cytokine staining (ICS) and by (B) enzyme‐linked immunosorbent assay (ELISA). (A, B) Data are shown as mean +SEM of (A) *n* = 51 and (B) *n* = 36 and are pooled from at least three separate experiments. *p*‐value derived by Mann–Whitney test. (C) Representative FACS plot and gating strategy for the phenotypic characterization of IL‐10 producing monocytes derived from healthy controls after LPS stimulation. All plots are gated on IL‐10^+^ CD14^+^ cells (denoted by asterisk). Numerical values refer to percentage of the asterisked gate.

We found that the proportion of IL‐10^+^ subset and the amount of IL‐10 produced were lower in monocytes from sarcoidosis patients compared to control, for a fixed number of monocytes (2.5 × 10^5^ cells) – median (IQR) = 11.6 (8.1–14.8)% of sarcoidosis monocytes versus 5.4 (4.4–7.6)% of control monocytes; *p* < 0.0001; and 25.6 (15.8–31.6) ng/mL versus 6.34 (4.8–10.0) ng/mL; *p* < 0.0001 (Fig. 1A and B). IL‐10‐expressing monocytes were CD14^+^CD16^−^CD206^−^CD115^+^CD15^−^ (Fig. 1C). We did not detect a unique identifying marker for these cells, and we did not detect a CD16^+^ monocyte population which expressed higher levels of IL‐10 after LPS stimulation.

### IL‐10 producing monocytes suppress T‐cell proliferation

We next questioned if IL‐10‐producing monocytes suppressed T‐cell proliferation. PBMCs were isolated from healthy individuals (*n* = 22) and CD14^+^ cells were first removed using CD14 MACS bead positive selection system to provide a monocyte‐free cell system for subsequent addition of fixed numbers of monocytes. These peripheral blood lymphocytes (PBLs) were then CFSE‐stained, and allogeneic monocyte‐derived DCs were added [1:^4^, DC:PBL]. Autologous CD14^+^ monocytes (generated using CD14 MACS beads negative selection) or CD19^+^ B cells as control (1:1, monocytes (or B cells):PBL) were then added. Monocytes and B cells were first prestimulated for 12 h with 1 mcg/mL of LPS, and washed before addition to the cell culture. Low intensity (CFSE^lo^) expression on CD3^+^ T cells on the fifth day was recorded to reflect the amount of proliferation in a given number of cells and period.

**Figure 2:**
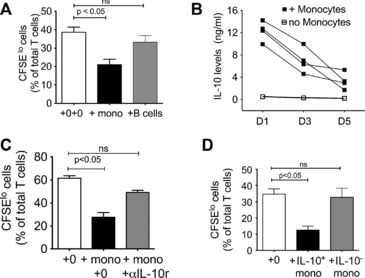
IL‐10‐producing monocytes suppress T‐cell proliferation. (A) Peripheral blood lymphocytes (PBLs) were isolated from healthy donors and stimulated with allogeneic PBLs in the presence of monocytes or B cells. Proliferation of CFSE‐labeled T cells was measured using flow cytometry. ‘‘0+0’’ refers to T‐cell proliferation without addition of monocytes. (B) The levels of IL‐10 in the supernatant of allogeneic T cells proliferating in the presence or absence of monocytes (as in (A)) were determined using ELISA on 1st, 3rd, and 5th day of culture. (C) Effect of addition of IL‐10 receptor (αIL‐10r) blockade on suppression of T‐cell proliferation by monocytes. Conditions identical to (A); αIL‐10r antibody was added at the start of the culture. ‘‘+ 0’’ refers to T‐cell proliferation without addition of monocytes. (D) Freshly isolated monocytes from healthy donors were enriched for IL‐10^+^ monocytes (IL‐10^+^ mono) or IL‐10^−^ cells (IL‐10^−^ mono), and the effect of these two monocyte fractions on T‐cell proliferation was assessed. Note that baseline proliferation varies among the independent assays — being lower in (D) than (C), so level of suppression referred to in text is calculated as percentage of baseline proliferation for that day. (A–D) Data are shown as mean + SEM of (A) *n* = 22 samples, (B, C) *n* = 5 donors and, (D) *n* = 9 donors and are representative of two‐three independent experiments. *p*‐values derived using Kuskal‐Wallis nonparametric multiple comparison test and Dunn's post hoc testing for (A) and (C); and one‐way ANOVA with Tukey's for post hoc tests for (D).

We found a significant reduction in T‐cell proliferation when monocytes were added which was not observed with B cells (Fig. 2A) (median (IQR) of 16.6 (11.9–24.9)% versus 34.6 (31.4–43.4)% of total T cells had divided i.e. CFSE^lo^ on 5th day). In order to determine the contribution of IL‐10 producing monocytes to suppression, we first measured IL‐10 levels on days 1, 3, and 5 of T‐cell proliferation and found that the levels were highest on day 1 (Fig. 2B). IL‐10 was not detected without addition of monocytes. IL‐10 receptor blockade (4 μg of LEAF™ purified anti‐human IL‐10R (CD210) antibody per 2 × 10^5^ PBLs) on days 1, 2, and 4 after initiation of culture restored the T‐cell proliferative capacity to levels observed without addition of monocytes (Fig. 2C), implying that IL‐10 was responsible for the T‐cell suppression. Although it was most likely that IL‐10 in this assay originated from monocytes, there was a theoretical possibility that leukocyte–monocyte interaction could generate IL‐10 producing lymphocytes. Since it was not possible to selectively block IL‐10 originating from monocytes in this system, we used MACS^TM^ IL‐10 capture beads to enrich the IL‐10‐producing fraction in the monocytes (from 18 to 55% of monocytes) before adding these to the cellular system. Monocytes depleted of IL‐10‐producing cells were used as controls. Addition of IL‐10‐enriched monocytes reduced T‐cell proliferation by nearly the same percentage (mean 38% reduction) as that when nonenriched monocytes were added (mean 47% reduction) (Fig. 2C) implying that nearly all the suppressive effect was accounted for by IL‐10 producing monocytes (median (IQR) of 30.2(27.2–42.90 versus 10.3(6.3–19.4)% of CFSE^lo^ T cells on 5th day). Suppression was not observed with addition of monocytes devoid of IL‐10^+^ cells (28.01(20.71–39.4)%) (Fig. 2D). Together, these in vitro findings support the notion that activated monocytes in the vicinity of activated T cells could control T‐cell proliferation via the production of IL‐10.

### Ability of monocytes to suppress T‐cell proliferation is defective in sarcoidosis

**Figure 3:**
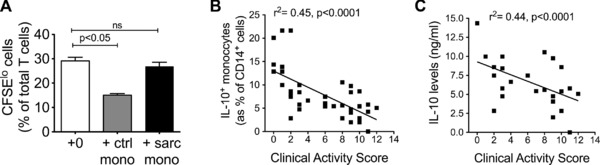
Monocytes derived from sarcoidosis patients fail to suppress T‐cell proliferation. (A) Peripheral blood lymphocytes (PBLs) were isolated from healthy donors and stimulated with allogeneic PBLs in the presence of T‐cell monocytes that were isolated either from sarcoidosis patients (‘‘+ sarc mono’’) (*n* = 7) or from healthy donors (‘‘+ctrl mono’’) (*n* = 7). Data are shown as mean + SEM and are representative of three independent experiments. Proliferation of CFSE‐labeled T cells was measured by flow cytometry. (B) The percentages of IL‐10‐producing monocyte in the peripheral blood of patients with sarcoidosis (as assessed by ICS), and (C) the levels of IL‐10 in the supernatant of LPS‐stimulated monocytes from patients with sarcoidosis (measured by ELISA), were plotted against disease activity as measured using a predefined composite score (Table 1); *n* = 40. Y‐axis in (B) and (C) refer to a fixed number of monocytes derived from each patient. *p*‐values were derived using Kuskal‐Wallis nonparametric multiple comparison test and Dunn's post hoc testing for (A); and Spearman's Rank test was used to assess correlation for (B) and (C).

We then questioned if this suppressive capacity is abnormal in monocytes derived from sarcoidosis patients. Using the same CFSE assay, we found that monocytes derived from sarcoidosis patients were unable to suppress T‐cell proliferation in contrast to that observed with monocytes derived from healthy controls (median (IQR) of 28.2(21.2–30.2) versus 31.6(24.0–31.6)% versus 15.0 (13.7–16.2) of CFSE^lo^ T cells on 5th day)(Fig. 3A).

We next asked if there were any ‘‘real‐time’’ correlation between the IL‐10 producing capacity of monocytes and the patient's level of clinical disease activity. Using a predefined, composite measure of clinical activity which included extent of chest radiograph abnormality, serum angiotensin converting enzyme, immunoglobulin levels and degree of peripheral lymphopenia (Table 1), 40 of the 51 patients were recruited spanning the entire predefined clinical activity score (0 to 12). We found that monocytes from patients exhibiting the lowest clinical disease activity score had the highest number of IL‐10 producing monocytes and the greatest capacity for IL‐10 production after LPS stimulation (Fig. 3B and C) (*p* < 0.0001; Spearman Rank test). This suggests a potential link in vivo between IL‐10 production by monocytes and clinical manifestation of disease, and indirectly with T‐cell activity and granuloma burden.

**Table 1:** Clinical activity score used to provide a measure of disease activity

CXR SCORE
*Abnormalities*
Normal = 0 Bilateral hilar lymphadenopathy or reticulation only (suggesting established fibrosis) = 1 Nodules or Consolidation +/− (ii) = 2
*Disease extent*
Score 1 for each CXR zone* affected (upper, mid, and lower zones; right and left)
CELLULAR SCORE
*Lymphocyte count* <1.0 × 10^9^/L = 1; >1.0 × 10^9^/L = 0
*Serum ACE* >100 U/L = 2; 55–100U/L = 1;<55 U/L (normal) = 0
*IgG* >13g/L = 1; </ = 13g/L = 0
Total maximum score = 12

*CXR ‘‘zones’’ refer to conventional radiographic definition — above anterior aspect of 2nd rib is upper zone; between anterior aspect of 2nd and 4th rib is mid zone and below 4th rib is lower zone.

### iNKT cells restore the T‐cell suppressive effect of monocytes from patients with sarcoidosis

**Figure 4:**
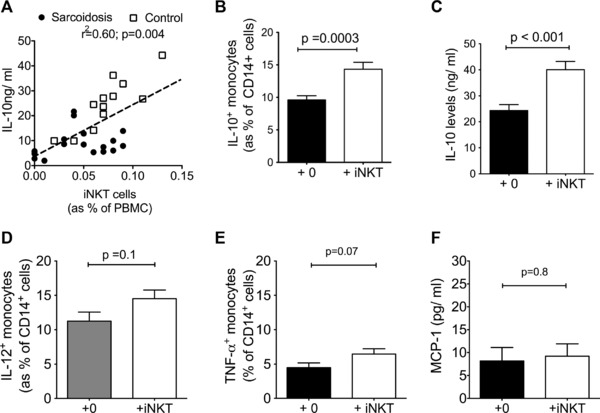
iNKT cells increase IL‐10 production by monocytes from healthy individuals. (A) Correlation of circulating iNKT‐cell numbers with IL‐10 levels in supernatant of LPS‐stimulated monocytes (18 h) from sarcoidosis patients (*n* = 18) and healthy controls (*n* = 12); at least three separate experiments (Spearman's Rank correlation test). Values shown are for correlation using all the data (i.e. sarcoidosis and controls). When values on cells from healthy donors and patients with sarcoidosis were analyzed separately, the correlation between healthy donor‐derived iNKT levels and IL‐10 from healthy donor‐derived monocytes was stronger (*r* ^2^ = 0.86; *p* < 0.0001), whereas there was no significant correlation between the two parameters for sarcoidosis patients. (B) Addition of iNKT cells to LPS‐stimulated monocytes from healthy individuals increased IL‐10 expression in monocytes, as determined by intracellular cytokine staining (ICS) at 18th h (monocytes from *n* = 36 healthy individuals). (C) IL‐10 levels in supernatant of cocultures from (B) in a subset (*n* = 22) of the cohort. (D, E) IL‐12 (*n* = 5) and TNF‐α (*n* = 20) expression was determined by ICS in monocytes from healthy donors that were stimulated with LPS in the presence of healthy donor‐derived iNKT cells. (F) MCP‐1 levels were determined in the supernatant of monocytes from healthy donors that were stimulated with LPS in the presence of healthy donor‐derived iNKT cells (*n* = 3). (B–F) Data are shown as mean + SEM and are representative of at least two experiments, apart from (F) where only one experiment was performed.

To explore if there were a relationship between the previously observed reduction in iNKT‐cell numbers and the defect in IL‐10 production by monocytes in sarcoidosis, we first examined the number of circulating iNKT cells with IL‐10 producing capacity of monocytes in sarcoidosis and normal controls. We found that the higher the numbers of circulating iNKT cells, the greater the capacity for IL‐10 production by monocytes when stimulated with LPS (*r* ^2^ = 0.6; *p* = 0.0004) (Fig. 4A) when all the data (i.e. sarcoidosis and controls, due to poor range in iNKT‐cell numbers in sarcoidosis patients). If these were analyzed separately, the correlation between normal iNKT levels and IL‐10 from monocytes was stronger (*r* = 0.86; *p* < 0.0001) while there was no significant correlation between the two parameters for sarcoidosis patients.

We then questioned whether human iNKT cells can influence monocyte function, specifically IL‐10 production, as this has not been reported before. Monocytes derived from 36 healthy individuals were cocultured with and without a CD4^+^ iNKT clone (LH22) (37) for 18 h in the presence of LPS (1 mcg/mL). Hereon all mention of ‘‘iNKT cells’’ refer to this LH22 iNKT clone. Different ratios of iNKT clone to monocytes (1:1, 1:2, and 1:4) were tested and IL‐10 levels were measured by both intracellular cytokine staining and ELISA for secreted protein in supernatant of fixed numbers of monocytes (2.5 ×10^5^ cells). For the first six samples, we found that addition of iNKT cells increased IL‐10 expression on monocytes (by intracellular cytokine staining (ICS)) with no significant difference between the ratios used; so we chose 1:1, iNKT: monocytes ratio to determine IL‐10 production for the rest of the 36 donors. We found a significant increase in both the number of IL‐10‐expressing cells (mean (SEM) of 14 (1.1) versus 9.6(0.7)% of circulating monocytes) and amount of IL‐10 production in the supernatant for LPS‐stimulated monocytes when co‐cultured with iNKT cells (mean (SEM) of 24.3(2.3) versus 40.1(3.1)ng/mL IL10 over 18 h) (Fig. 4B and C). By ICS, we showed that only CD14^+^ cells (monocytes) in the coculture expressed IL‐10, implicating these cells as the sole source of IL‐10 in the supernatant. To examine the effect of iNKT cells on other monocytic function, we examined LPS‐induced IL‐12 and TNF‐α expression by monocytes with or without coculture with iNKT cells; and measured MCP‐1 levels in supernatant. In contrast to IL‐10 production, we found that iNKT cells had no effect on production of these cytokines and chemokine (Fig. 4D–F).

**Figure 5:**
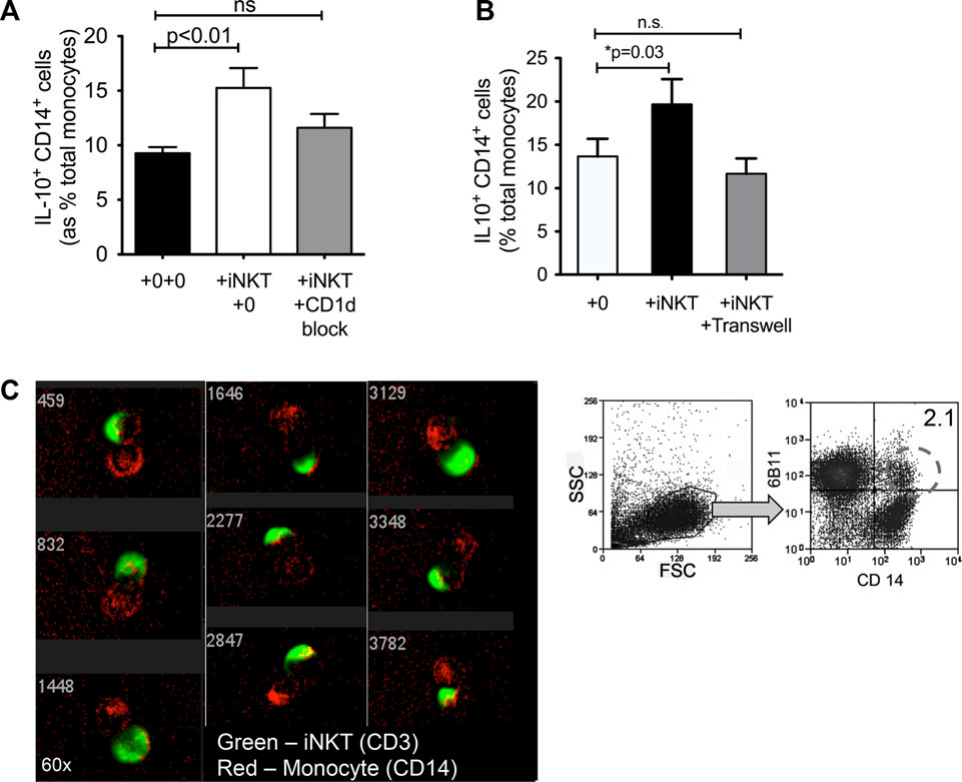
iNKT cells increase IL‐10 production by monocytes after in a cell contact‐dependent manner. (A) Monocytes and iNKT cells from healthy donors were cocultured in the presence or absence of CD1d‐blocking antibody, and IL‐10 expression by monocytes was measured by ICS after 18 h of stimulation with LPS (*n* = six healthy donors; two separate experiments). (B) Monocytes and iNKT cells from healthy donors were cocultured either separated by a transwell or in a conventional culture plate and stimulated for 18 h with LPS. IL‐10 expression was analyzed by ICS. (A, B) Data are shown as mean + SEM of *n* = 6 healthy donors and are representative of two independent experiments. (C) CD14^+^6B11^+^ single cells gated as shown in the FACS dot plot on right were imaged using ImageStream^TM^ technology (*n* = one experiment). Imaging showed CD3^+^ iNKT cells (green) and CD14^+^ cells (red) interacting with each other. Numbers refer to cell number — 5000 consecutive single cells were analyzed from this gate. Figure shows a randomly selected image of nine interacting cells captured from this analysis.

To determine if CD1d engagement was required for production of IL‐10 from monocytes, we pulsed monocytes with 25 mcg/mL CD1d blocking antibody (CD1d41.2) (dose optimized for maximal functional blocking in previous studies) 26 before coculture and found that this significantly reduced IL‐10 expression, although did not return the levels to baseline (Fig. 5A). However, with complete physical separation by Transwell, the increased IL‐10 production generated by iNKT cells was abolished (Fig. 5B). This suggests that direct physical signals such as adhesion and costimulatory molecules are likely to play a role in ‘‘licensing’’ monocytes to produce IL‐10 when interacting with iNKT cells. Using ImageStream^TM^ analysis, which allows single‐cell visualization and phenotyping, we found that at any one point, 2% of cells are in contact with each other as doublets, regardless of the ratio of iNKT to monocytes (Fig. 5C, right panel). By direct visualization, these doublets were single iNKT cells in direct contact with another monocyte (rather than nonspecific aggregation of cells) (Fig. 5C, left panel).

**Figure 6:**
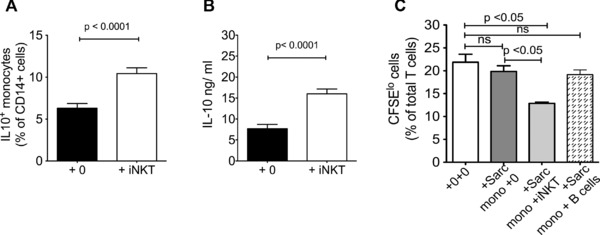
iNKT cells from healthy donors restore IL‐10 production and T‐cell suppressive activity of monocytes derived from sarcoidosis patients. (A, B) Addition of iNKT cells significantly increased IL‐10 production after 18 h of LPS stimulation in monocytes from sarcoidosis patients. (A, B) Data are shown as mean + SEM of (A) *n* = 33 and (B) *n* = 25 samples and are representative of at least three independent experiments. (C) Peripheral blood lymphocytes (PBLs) were isolated from patients with sarcoidosis and stimulated with allogeneic PBLs in the presence of monocytes from patients with sarcoidosis in the presence or absence of iNKT cells. Proliferation of CFSE‐labeled T cells was measured using flow cytometry T‐cell. Monocytes from sarcoidosis patients could suppress T‐cell proliferation when iNKT cells were added to the coculture at a 1:1 monocyte:iNKT‐cell ratio. Data are shown as mean + SEM of *n* = 7 sarcoidosis donors and are representative of two independent experiments.

Finally we show that addition of iNKT cells to monocytes derived from sarcoidosis patients restored the proportion of IL‐10‐producing cells to that observed in healthy monocytes (Fig. 6A) (median (IQR) of 5.3(4.3–8.4)% to 10.3(8.0–11.4)% of monocytes, and increased the levels of IL‐10 in the supernatant of these cocultures (Fig. 6B) (6.0(4.2–10) to 15.3(12.9–18.9) ng/mL). When iNKT cells (but not B cells) were added to the CFSE assay (as described in Fig. 3A), the suppressive capacity of the sarcoidosis monocytes was also restored to levels observed for monocytes from healthy controls (Fig. 6C).

## Discussion

Our study shows that monocytes from patients with sarcoidosis do not produce IL‐10 to the same level as healthy controls and are functionally less capable of suppressing T‐cell proliferation. It also suggests a potential link between this defect and the previously observed reduction in numbers of iNKT cells in this condition.

One of the strengths of the study is the use of careful isolation methods to ensure a pure and unstimulated population of monocytes from patients and healthy controls. Monocytes are easily activated by even relatively inert materials such as Ficoll used for density gradient isolation 28. The methods we employed ensure closest correlation between ex vivo findings and in vivo processes. Another significant aspect of the study is our patient cohort, which comprised never‐smokers and treatment‐naïve patients, and the use of a composite method to measure disease activity. These measurements give a defined and standardized quantification of disease activity where no validated universal score currently exists.

Several hypotheses exist for the aetio‐pathology of sarcoidosis, including autoimmune response to an uncovered or cross‐reactive self‐epitope [e.g. 29], loss of self‐tolerance and reduced rate of T‐cell apoptosis. The most compelling is probably Moller's hypothesis which implicates loss of T‐cell control during the priming phase as a critical factor in disease genesis 8. Our data supports this, and could also explain the ‘‘immunologically exhausted’’ nature of FoxP3 Treg cells observed in sarcoidosis 16 since these cells are functioning without the contribution from IL‐10 producing monocytes. As monocytes are early players in an immune response, we suggest that this failure to produce IL‐10 and exert a regulatory check on T‐cell proliferation represent a ‘‘check‐point’’ failure, which contributes to heightened T‐cell activity and granulomagenesis.

The ability of iNKT cells to influence IL‐10 production by monocytes has not been demonstrated before, although interactions between iNKT cells and macrophages and monocyte‐derived DCs have been shown. The closest study, from Hedge et al., showed that IL‐4 and GM‐CSF conditioned monocyte (therefore monocyte‐derived DCs) cocultured with iNKT cells produced IL‐10 (but not IL‐12) 30, corroborating our findings on nonconditioned monocytes. Together, our findings suggest that iNKT cells might ‘‘license’’ monocytes to specifically produce IL‐10. One interesting aspect of our finding is that iNKT cells were able to increase monocytic IL‐10 levels without exogenous glycolipid like α‐galactosyl ceramide which has been widely used to specifically activate iNKT cells. Therefore, the ability to increase IL‐10 production depends on signals generated by presentation of a self/endogenous‐antigen on CD1d. However it is likely that other paths of cross talk occur to enhance this endogenous stimulation since optimal CD1d blockade caused reduction but not abrogation of IL‐10 production. We also acknowledge that the studies were performed with iNKT clones which may put some constraints on the ability to generalize the findings to iNKT cells in general.

The question arises as to where this interaction between iNKT cells and monocytes might occur. Although one possibility is the blood, the sheer force of movement of circulating cells makes this unlikely. In addition, the frequency of iNKT cells in humans is very small. Because there needs to be physical contact between iNKT cells and monocytes for the iNKT cells to influence IL‐10 production, it is more likely that this occurs in spleen, lymph node or bone marrow, where monocytes and iNKT cells are brought together for a longer period of time. We propose that the most likely sites are lymph nodes and spleen 31, where the consequence of iNKT and monocyte interaction can have an immediate impact on T‐cell proliferation. Our in vitro studies suggest that only a small numbers of cells (2%) need to be in contact at any point for the interaction to cause an increase in IL‐10 production.

This study was driven, in part, by our interest and previous findings in the interaction between iNKT cells and monocytes. Thus, we have not examined the potential effect of iNKT cells on other CD1d‐expressing, IL‐10‐producing cells such as DCs and regulatory B cells. A recent study showed that IL‐10 producing B cells (or ‘‘B reg cells’’) were increased in sarcoidosis 17, resonating the findings of increased FoxP3 Treg cells in this condition 16. It has been suggested that this increase is a response to the high T‐cell activity. Although all IL‐10 producing cells are likely to have T‐cell suppressive activity, IL‐10 monocytes are unique in its temporal position in sarcoidosis disease pathogenesis — they come into play much earlier, and we postulate that failure of monocytes to control T‐cell activity contributes to T‐cell hyperactivity which then cause a secondary increase in Treg cells and B reg cells. In addition, monocytes are precursors to tissue macrophages and granuloma, so abnormalities in these cells co‐localizes T‐cell hyperactivity to the site of granulomagenesis. Our study does not provide a mechanism by which iNKT cells might influence IL‐10 production in monocytes. However, it seems clear that iNKT cells increased the numbers of IL‐10 producing monocyte, therefore iNKT cells may directly induce IL‐10 signaling pathways. Several routes are possible 32. One hypothesis is that iNKT cells act via a TLR‐independent route, for example, Dectin‐1, a C‐type lectin which has been shown to promote IL‐10 production upon its ligation 33. Indeed, we have shown that iNKT cells can increase Dectin‐1 levels on murine monocytes 27.

Notwithstanding the lack of a molecular mechanism for iNKT‐directed IL‐10 increase in monocytes, we suggest that under baseline circumstances, iNKT cells are involved in contacting and interacting with monocytes to impart a signal which heightens or induces the ability of monocytes to produce IL‐10 when they encounter pathogens via their pattern recognition receptors (i.e. TLR4). Monocytes are then involved in regulating the ensuing adaptive immune (T cell) response and ensuring a measured response when activated. In the absence of this regulatory pathway, T‐cell activity proceeds unchecked. As in most complex polygenic disease, the defect in IL‐10 production by monocytes is likely to be a contributory rather than sole mechanism in pathogenesis.

These findings make monocytic functions and pathways potential new therapeutic targets in sarcoidosis. This is particularly exciting because these immune pathways and characteristics are now well characterized and monocytes, being circulating rather than sequestered cells can be easily targeted in the blood and changes tracked in blood tests. Indeed this pathway is targeted in other diseases, and the major homing receptor (CC‐chemokine receptor 2 or CCR2) for monocytes which responds to the chemokine, MCP‐1 is already the target of CCR2 antagonists drugs which are already in clinical trials 34.

Although the focus of the study is on IL‐10 producing monocytes, the reduction of iNKT cells in sarcoidosis patients may be worth a mention. Since we published the observation of low or absent iNKT cells in blood, lung lavage, and draining lymph nodes of sarcoidosis patients using αGC‐loaded tetramers and Vα24Vβ11 TCR mAbs 23, further work has confirmed that these cells are indeed low and its residual numbers are inversely correlated with exaggerated BALF lymphocytosis and CD4^+^ T‐cell responses 24, supporting our proposition that deficiency in iNKT numbers is linked to excessive T‐cell proliferation. In addition, residual iNKT cells display markers of functional exhaustion and loss of IFN‐γ production 35, indicating that even with the small numbers remaining, the cells are deficient functionally.

In conclusion, our study shows that IL‐10 production and T‐cell regulation capabilities by monocytes are defective in patients with sarcoidosis. Our in vitro data suggest this could be secondary to loss of iNKT cells, which under normal circumstances enhanced monocytic IL‐10 production. These findings offer new insights into the cause for T‐cell overactivity in sarcoidosis and provide fresh platforms for therapeutic exploration in sarcoidosis.

## Materials and methods

### Subjects

Sarcoidosis patients were recruited from the Oxford Sarcoidosis Clinic, Oxford Centre for Respiratory Medicine. All patients were diagnosed according to the American Thoracic Society and WASOG Statement for Sarcoidosis 36. Inclusion criteria were (i) never‐smoker, (ii) not on treatment at the point of sampling and (iii) no concomitant disease. All patients had histology confirmation of diagnosis. Disease activity was scored using a predefined composite scoring system that integrates serum angiotensin converting enzyme (sACE) levels, immunoglobulin (IgG) levels, presence of lymphopenia and extent of chest X‐ray abnormalities (Table 1).

Healthy controls were recruited through local advertising in accordance with national ethical approval. All healthy controls were assessed by a physician and had no medical conditions. All subjects gave informed consent and the study was approved by the National Research Ethics Committee. Overall demographics and clinical characteristics where patients were compared to controls are shown in Table 2.

**Table 2:** Characteristics of subjects for studies comparing sarcoidosis and healthy controls

Figure	Fig 1A and 3B		Fig 1B and 3C		Fig 4A	
Subjects	S	HC	S	HC	S	HC
Total subjects, *n*	51	25	36	12	18	12
Total events	57	25	40	12	18	12
Mean age (SD) in years	49.9 (12.7)	41.0(11.0)	48.2(10.6)	42.0(13.0)	47.9(8.0)	35.0(7.0)
CXR stage 0/1/2/3/4^a^	15/11/15/11/15	NA	8/7/11/10/4	NA	5/5/3/3/2	NA

S, sarcoidosis; HC, healthy controls. NA, not applicable (where there are no controls).

a Scadding CXR stage: 0 = no adenopathy, no lung infiltrates; stage 1, hilar adenopathy only; stage 2, hilar adenopathy and lung infiltrates; stage 3, lung infiltrates only; stage 4, pulmonary fibrosis. Last row refers number of patients per each Scadding stage. SD, standard deviation.

### Cells and cellular assays

All assays were performed on freshly isolated cells. For the isolation of purified, ‘‘untouched’’ monocytes, cells were negatively selected using magnetic MACS MicroBeads (Pan Monocyte isolation kit) (Miltenyi Biotech, Bisley, U.K.) according to manufacturer's protocol. We optimized this kit with Miltenyi in order to include CD16^+^CD14^+^ monocytes (the original kit had CD16 mAbs which meant that CD16^+^ cells including CD14^+^CD16^+^ monocytes would have been depleted). Frequency of iNKT cells was determined using 6B11 mAb which recognizes an epitope on the CDR3 region of the Vα24 TCR on iNKT cells. Previous and recent validation studies have shown that 6B11 staining reflected αGC‐loaded CD1d tetramer staining 23.

Pure populations of iNKT cells (LH22) were derived by limiting dilution cell cloning from a healthy individual 37. All mentions of iNKT cells in the text refer to this iNKT clone.

### Flow cytometric studies

All mAbs were from eBiosciences (Hatfield, U.K.). Flow cytometry and intracellular cytokine staining protocols have been previously described 22. To exclude dead cells, cells were also stained with 1 in 1000 dilution of 10 mg/mL Hoechst (Sigma) for 15 min at room temperature before washing in FACS buffer. Imagestream analysis were performed on Amnis Imagestream^TM^ IS100 flow cytometer (Seattle, USA) using IDEAS® analytical software (https://www.amnis.com/imagestream.html).

### ELISA

IL‐10 ELISA was performed using Biotin anti‐human and anti‐viral IL‐10 antibody kit (BD Biosciences, Oxford, U.K.) and avidin‐horse radish peroxidase. Plates were read on a microplate reader (Bio‐Rad, Model 680). All IL‐10 levels were obtained from supernatant, which were not subjected to Brefeldin incubation.

### IL‐10 capture assay

IL‐10 producing monocytes were enriched/ depleted using MACS IL‐10 secretion assay, enrichment and detection kit (Miltenyi Biotec) according to manufacturer's protocol.

### Statistics

All statistical analysis was performed using GraphPad Prism 5.01 (GraphPad, La Jolla, USA). Normality of data distribution was first tested using the D'Agostino & Pearson omnibus normality test. Normally distributed data sets were compared using Student's *t*‐test; where comparisons included data not normally distributed, the Mann–Whitney test was used to compare data sets. To compare multiple, normally distributed paired data sets simultaneously, one‐way ANOVA with Tukey post‐test analysis was used. Where multiple data sets included data that were not normally distributed, Kruskal‐Wallis with Dunn's multiple comparison post‐test analysis was performed. Spearman's Rank test was used to assess correlation. A *p*‐value of less than 0.05 was considered significant. Mean values were reported for normally distributed data and median for nonnormal distribution. Error bars on graphs represent SEM unless otherwise stated.

## Conflict of interest

The authors declare no commercial or financial conflict of interest.

**Abbreviations**
CD1d: cluster differentiation 1d
IgG: Ig G
iNKT: invariant natural killer T cell
IQR: interquartile range

## Supporting information

As a service to our authors and readers, this journal provides supporting information supplied by the authors. Such materials are peer reviewed and may be re‐organized for online delivery, but are not copy‐edited or typeset. Technical support issues arising from supporting information (other than missing files) should be addressed to the authors.

Peer review correspondence
