# Supplementary material for: Abnormalities in iNKT cells are associated with impaired ability of monocytes to produce IL‐10 and suppress T‐cell proliferation in sarcoidosis
Source: Eur J Immunol. 2014 May 12;44(7):2165–74. doi: 10.1002/eji.201344284 (PMC4746743; doi:10.1002/eji.201344284)
Supplement: Supplementary file 1 — Peer review correspondence [file EJI-44-2165-s001.pdf]

Peer review correspondence

European Journal of Immunology

Abnormalities in iNKT cells are associated with impaired ability of monocytes to produce IL-10 and suppress T-cell proliferation in sarcoidosis Anjali Crawshaw, Yvonne R Kendrick, Andrew J McMichael and Ling-Pei Ho

Corresponding author: Dr Ling-Pei Ho, Weatherall Institute of Molecular Medicine, University of Oxford, Oxford OX3 9DS, UK.

Review Timeline:	Submission date:	12 November 2013
	First Editorial decision:	20 December 2013
	First Revision received:	19 February 2014
	Accepted:	2 April 2014

Handling Executive Committee member: Prof. in McInnes

Please note that the correspondence below does not include the standard editorial instructions regarding preparation and submission of revised manuscripts, only the scientific revisions requested and addressed.

First Editorial Decision – 20 December 2013

Dear Dr. Ho,

My apologies for the slight delay in processing the review of your manuscript ID eji.201344284 entitled "Impaired monocytic IL-10 production in sarcoidosis and potential link to abnormalities in iNKT cells" which you submitted to the European Journal of Immunology. One referee report was severely delayed but now all opinions have been received and the comments of the referees are included at the bottom of this letter.

A revised version of your manuscript that takes into account the comments of the referees will be reconsidered for publication.

You should also pay close attention to the editorial comments included below. *In particular, please edit your figure legends to follow Journal standards as outlined in the editorial comments. Failure to do this will result in delays in the re-review process.*

Please note that submitting a revision of your manuscript does not guarantee eventual acceptance, and that your revision will be re-reviewed by the referees before a decision is rendered.

If the revision of the paper is expected to take more than three months, please inform the editorial office. Revisions taking longer than six months may be assessed by new referee(s) to ensure the relevance and timeliness of the data.

Once again, thank you for submitting your manuscript to European Journal of Immunology and we look forward to receiving your revision.

Yours sincerely,

European Journal of Immunology

Peer review correspondence

Karen Chu

On behalf of Prof. Iain McInnes

Dr. Karen Chu
Editorial Office
European Journal of Immunology
e-mail: ejied@wiley.com
www.eji-journal.eu

****

Reviewer: 1

Comments to the Author
The manuscript is very interesting but it needs some improvements:

Important question is: if the Authors repeat the same study using CD14+CD16+ monocytes, they will obtain a low level of Il-10? Probably not. It is well known that in contrast to CD14+CD16+ monocytes, CD14+CD16- produces a low level of IL-10.

1. Regarding the Abstract section:

a) From this text we don't know, where iNKT and IL-10 concentration were tested, in a blood or BAL?

2. Regarding the Introduction section:

a) Authors should describe a role of iNK-cells in the context of immunity, especially in sarcoidosis.
b) Authors should write about immune response in blood and in lungs because there is an opposite immunological response in peripheral blood and in the lung.
c) IL-10 level in a peripheral blood from SA patients is increased in many studies, why in the current study it was decreased? In some studies, a low production of IL-10 was present also in TB.We know that increased and chronic TNF-α and IL-6 production induces a secretion of anty-proinflammatory IL-10.

3. Regarding material and methods section:

a) What about homogeneity (extrapulmonary SA and race) of SA patients?
b) What about TB examination?

4. Regarding the discussion section:

a) What about an autoimmunity, suggested by Dubaniewicz et al in the etiopathogenesis of sarcoidosis? What about a self-tolerance loss? what about an apoptosis of T-cells?
b) What about phenotypes of monocytes in peripheral blood and lungs? They circulate and they are changed during transmigration blood-lungs (Dubaniewicz et al. Hum Immunol. 2012 Aug;73(8):788-94, Strauss-Ayali D, Conrad SM, Mosser DM. Monocyte subpopulations and their differentiation patterns during infection. J Leukoc Biol 2007;82:244–52. Alexis N, Soukup J, Ghio A, Becker S. Sputum phagocytes from healthy individuals are functional and activated: a flow cytometric comparison with cells in bronchoalveolar lavage and peripheral blood. Clin Immunol 2000;97:21–32.)
c) Why have the Authors tested subset of monocytes CD14+CD16-CD206-CD115+CD15-, not non-classical CD14+CD16+, which increased occurrence is characteristic for sarcoidosis and produces increased level of IL-10? Maybe it is a reason of obtained low level of IL-10.
d) The Discussion section is too long and it is not concrete; there are a lot hypotheses

Reviewer: 2

European Journal of Immunology

Comments to the Author
Major comments:
1. Based on the methods section, there appears to be an important typo in the second paragraph of the results section where there is reference to “negative selection” of monocytes. Please clarify as whether the study involved negative or positive selection seems critical to this study……the text in question is as follows: “We next questioned if IL-10-producing monocytes suppressed T cell proliferation. PBMCs were isolated from healthy individuals (n=12) and CD14+ cells were first removed using CD14 MACS bead positive selection system to provide a monocyte-free cell system for subsequent addition of fixed numbers of monocytes. These peripheral blood lymphocytes (PBL) were then CFSE-stained, and allogeneic monocyte-derived DCs were added [1:4, DC:PBL]. Autologous CD14+ monocytes (generated using CD14 MACS beads negative selection) or CD19+ B cells as control [1:1, monocytes (or B cells):PBL] were then added.”

2. This reviewer has concerns regarding the authors’ composite score. CXR manifestations in sarcoidosis connote severity of disease, not necessarily activity of disease. BAL lymphocytosis is an accepted measure of disease “activity”. Also, the authors’ composite measurement has not been validated which they state in the text: “no validated universal score currently exists” so why do the authors think that their score/measurement is the “best”? Further, how did the authors arrive at the score cut offs between low and high disease activity? Why not use the median value instead?

3. The text in the results section for Fig 4A is misleading in current form and should state what is stated in the legend. Specifically, that there is NO correlation between iNKT cell frequencies vs. IL10 concentration in sarcoidosis subjects.

4. In the discussion, the authors should acknowledge the use of an NKT cell clone and its limitations in generalizing the findings without replication with ex vivo iNKT cells.

Minor comments:
1. Consider changing to TNF–α and IFN–γ rather than what appears in the text which is TNF –a and IFN–g when referring to TNF and IFN

2. Should refer to the iNKT cell clone as “iNKT cell clone” throughout the appropriate sections of the manuscript instead of referring to them as “iNKT cells”. This can be confusing to the reader as to whether you used iNKT cells directly ex vivo from subjects or a iNKT cell clone.

3. This paragraph is slightly confusing. Please try to clarify the findings: “Finally, we show that addition of iNKT cells [clones correct?] (at 1:1 monocyte to iNKT ratio) to monocytes [from sarcoidosis subjects, and isolated by negative selection?] restored (i) [the] number of IL-10-producing cells in the co-culture, (ii) IL-10 levels to near that observed for monocytes isolated from healthy controls [Figure 6a-b; median (IQR) of 5.3(4.3-8.4)% to 10.3(8.0-11.4)% of monocytes, and 6.0(4.2-10) to 15.3(12.9-18.9)ng/ml] and (iii) the T cell suppressive capacity to that of non-sarcoidosis/healthy monocytes (Figure 6c).

4. Table 2 states that Fig 2A has 7 sarcoid subjects and no HC. But it seems that related results text section reports 12 HC used for the results in Fig 2A and the legend reports 15 HC. Please clarify. Also, the authors created a nice format (table 2) to present the different sample sizes for SOME of the figures, but not ALL of the figures. Why is this? Perhaps making it consistent would make it A LOT easier to follow (either put all the sample sizes for all figures in the table or get rid of the table and put them in the text, or the figure legends?)

5. On pg 10 of results, the authors state that “only CD14+ cells (monocytes) in the co-culture expressed IL-10, implicating these cells as the sole source of IL-10 in the supernatant”. But didn’t the experimental design consist of sorting out CD14+ monocytes from 36 controls and culturing them with NKT clones? So are you saying that the NKT clone was not the source of IL-10? If this is not correct, please considering clarifying the sentence.

6. No mention in the statistical section of the manuscript about what type of correlation plot was used.

7. Is reference #35 correctly cited in the text related to NKT cell clones?

8. Figure legend 3B. This is not technically a correlation

<u>First Revision – authors' response – 6 December 2013</u>

Point-by-point response

Reviewer: 1

Comments to the Author
The manuscript is very interesting but it needs some improvements:
Important question is: if the Authors repeat the same study using CD14+CD16+ monocytes, they will obtain a low level of Il-10? Probably not. It is well known that in contrast to CD14+CD16+ monocytes, CD14+CD16- produces a low level of IL-10.

The study was performed by MACS bead CD14 negative selection kit. This kit isolates CD14+ cells without touching CD14+ cells. We optimised this kit with Miltenyi in order to include CD16+CD14+ monocytes (the original kit had CD16 mAbs which meant that CD16+ cells including CD14+CD16+ monocytes would have been depleted). Thus our monocytes contained both CD16+ and CD16- populations. Hardly any of the CD16+ monocytes produced IL-10 after LPS stimulation (we showed this in Figure 1c). These are all freshly isolated monocytes used immediately (ie not thawed from frozen). We have made this clearer in view of this reviewer's comments.

1. Regarding the Abstract section:
a) From this text we don't know, where iNKT and IL-10 concentration were tested, in a blood or BAL?

Apologies, these are from blood – this is now in the abstract.

2. Regarding the Introduction section:
a) Authors should describe a role of iNKT-cells in the context of immunity, especially in sarcoidosis.

Thank you – have done now (page 5).

b) Authors should write about immune response in blood and in lungs because there is an opposite immunological response in peripheral blood and in the lung.

Yes, agree – we didn't put it in originally to keep the introduction succinct and targeted but we agree it would be informative. Now added in (page 3).

c) IL-10 level in a peripheral blood from SA patients is increased in many studies, why in the current study it was decreased? In some studies, a low production of IL-10 was present also in TB. We know that increased and chronic TNF-α and IL-6 production induces a secretion of anty-proinflammatory IL-10.

Most studies refer to IL-10 levels in bronchoalveolar lavage and even then the levels were low, and almost no studies compared sarcoidosis patients with healthy controls (mainly between acute and chronic). Data on IL-10 in peripheral blood in sarcoidosis is conflicting with some reports of it being reduced and others increased when measured directly in whole blood or serum in which case, the source of the IL-10 is unclear/ multiple. We are not commenting on serum IL-10 levels, rather the IL-10 production by a subset cells (monocytes) in the peripheral blood.

3. Regarding material and methods section:
a) What about homogeneity (extrapulmonary SA and race) of SA patients?

Thank you. Of the 51 patients, 2 were African-Caribbean, 3 South Asian (India/ Pakistan) and the rest were Caucasians. All were non-smokers. All had pulmonary involvement, 1 had lung, heart, uveitis, 5 had lung and skin, 12 had lung and uveitis, 2 had lung and cardiac sarcoidosis. We have detailed this now in the first part of Results.

b) What about TB examination?

All patients had bronchoalveolar lavage at presentation with samples sent to microbiology laboratory to exclude infectious organs, specifically including TB. We have detailed this now in the first part of Results.

4. Regarding the discussion section:

a) What about an autoimmunity, suggested by Dubaniewicz et al in the etiopathogenesis of sarcoidosis? What about a self-tolerance loss? What about an apoptosis of T-cells?

All possible. We have mentioned these briefly now and included Dubaniewicz's reference to autoimmunity.

b) What about phenotypes of monocytes in peripheral blood and lungs? They circulate and they are changed during transmigration blood-lungs (Dubaniewicz et al. Hum Immunol. 2012 Aug;73(8):788-94, Strauss-Ayali D, Conrad SM, Mosser DM. Monocyte subpopulations and their differentiation patterns during infection. J Leukoc Biol 2007;82:244–52. Alexis N, Soukup J, Ghio A, Becker S. Sputum

European Journal of Immunology

phagocytes from healthy individuals are functional and activated: a flow cytometric comparison with cells in bronchoalveolar lavage and peripheral blood. Clin Immunol 2000;97:21–32.)

Thank you. We were particularly interested in the monocytes in the circulation and not in the BAL because we were focusing on the upstream behaviour of monocytes before these cells migrate to the lungs or other organs. This is to provide us with a means of potentially addressing altered behaviour in these monocytes before they differentiate into macrophage or contribute to granuloma formation. The phenotype of monocytes in the peripheral blood was extensively studied, with particular focus on identifying surface markers/ other phenotypic characteristics for the IL-10 producing subset. None of the following expression: CD11b, CD11c CD14, CD15, CD16, CD32, CD62l, CD115, CD163, CCR2, CX3CR1 and HLADR were increased or reduced in the IL-10 producing subset (Figure 1c).

c) Why have the Authors tested subset of monocytes CD14+CD16-CD206-CD115+CD15-, not non-classical CD14+CD16+, which increased occurrence is characteristic for sarcoidosis and produces increased level of IL-10? Maybe it is a reason of obtained low level of IL-10.

We have tested both CD14+CD16+ and CD14+CD16-, ofcourse! They are in the same panel of mAbs we used to examine whether the there are any markers that would help us identify the IL-10 producing monocytes (see figure 1c). Sorry this is not clear – we have made it clearer by explaining that we have used these mAbs to determine it the IL-10 expressing monocytes expressed any of these markers. As per response to comment (1), hardly any of the CD16+ monocytes produced IL-10 after LPS stimulation.

d) The Discussion section is too long and it is not concrete; there are a lot of hypotheses
Agree – have cut down.

Reviewer: 2
Comments to the Author
Major comments:
1. Based on the methods section, there appears to be an important typo in the second paragraph of the results section where there is reference to “negative selection” of monocytes. Please clarify as whether the study involved negative or positive selection seems critical to this study……the text in question is as follows: “We next questioned if IL-10-producing monocytes suppressed T cell proliferation. PBMCs were isolated from healthy individuals (n=12) and CD14+ cells were first removed using CD14 MACS bead positive selection system to provide a monocyte-free cell system for subsequent addition of fixed numbers of monocytes. These peripheral blood lymphocytes (PBL) were then CFSE-stained, and allogeneic monocyte-derived DCs were added [1:4, DC:PBL]. Autologous CD14+ monocytes (generated using CD14

MACS beads negative selection) or CD19+ B cells as control [1:1, monocytes (or B cells):PBL] were then added."

For this sentence '..We next questioned if IL-10-producing monocytes suppressed T cell proliferation. PBMCs were isolated from healthy individuals (n=12) and CD14+ cells were first removed using CD14 MACS bead positive selection system to provide a monocyte-free cell system for subsequent addition of fixed numbers of monocytes…', the CD14+ cells have to be removed from the PBMC in order that the there are no monocytes in these cells (the 'monocyte-free cell system'). This necessarily **has** to be done by CD14 positive selection. These monocytes are then discarded. Then, to add in a controlled and standard number of monocytes, we derived allogeneic monocytes using CD14 negative selection and added this into the monocyte-free cell system, as in this part of the statement "…Autologous CD14+ monocytes (generated using CD14 MACS beads negative selection) or CD19+ B cells as control [1:1, monocytes (or B cells):PBL] were then added."

2. This reviewer has concerns regarding the authors' composite score. CXR manifestations in sarcoidosis connote severity of disease, not necessarily activity of disease. BAL lymphocytosis is an accepted measure of disease "activity". Also, the authors' composite measurement has not been validated which they state in the text: "no validated universal score currently exists" so why do the authors think that their score/measurement is the "best"? Further, how did the authors arrive at the score cut offs between low and high disease activity? Why not use the median value instead?

Yes, apologies - in retrospect we agree that we should not have used the word 'best' because indeed validation of this has not been published. We have however, since validated this against BAL lymphocytosis, sol-IL2r levels, and abnormalities on high resolution CT scan; but this has not been published yet. So, we have changed the word 'best' to ' a defined and standardized' quantification of disease activity. The high and low score cut offs were arbitrary. We have now put in the whole range of disease activity score in order to remove the uncertainties over the definition of high and low activity. This showed a highly significant correlation between proportion of IL-10+ monocytes (and IL-10 levels in culture supernatant) and the disease activity score.

3. The text in the results section for Fig 4A is misleading in current form and should state what is stated in the legend. Specifically, that there is NO correlation between iNKT cell frequencies vs. IL10 concentration in sarcoidosis subjects.

Thank you, we have changed it to what it says in the legend.

European Journal of Immunology

4. In the discussion, the authors should acknowledge the use of an NKT cell clone and its limitations in generalizing the findings without replication with ex vivo iNKT cells.

Thank you, we have done so now in Discussion.

Minor comments:

1. Consider changing to TNF–α and IFN–γ rather than what appears in the text which is TNF –a and IFN–g when referring to TNF and IFN

Apologies – this was an oversight and we have gone through this carefully now.

2. Should refer to the iNKT cell clone as “iNKT cell clone” throughout the appropriate sections of the manuscript instead of referring to them as “iNKT cells”. This can be confusing to the reader as to whether you used iNKT cells directly ex vivo from subjects or a iNKT cell clone.

OK – agree. As iNKT cell clone does not sound quite right, we have specified in the Methods and at the beginning of Results that all reference to iNKT cells refer to the iNKT LH22 clone.

3. This paragraph is slightly confusing. Please try to clarify the findings: “Finally, we show that addition of iNKT cells [clones correct?] (at 1:1 monocyte to iNKT ratio) to monocytes [from sarcoidosis subjects, and isolated by negative selection?] restored (i) [the] number of IL-10-producing cells in the co-culture, (ii) IL-10 levels to near that observed for monocytes isolated from healthy controls [Figure 6a-b; median (IQR) of 5.3(4.3-8.4)% to 10.3(8.0-11.4)% of monocytes, and 6.0(4.2-10) to 15.3(12.9-18.9)ng/ml] and (iii) the T cell suppressive capacity to that of non-sarcoidosis/healthy monocytes (Figure 6c).

Yes it is rather confusing - apologies, and thank you for pointing this out. We have changed it to such – Finally we show that addition of iNKT cells to monocytes derived from sarcoidosis patients restored the proportion of IL-10 –producing cells to that observed in healthy monocytes (Figure 6a) [median (IQR) of 5.3(4.3-8.4)% to 10.3(8.0-11.4)% of monocytes], and increased the levels of IL-10 in the supernatant of these co-cultures (Figure 6b) [6.0(4.2-10) to 15.3(12.9-18.9)ng/ml]. When iNKT cells (but not B cells) were added to the CFSE assay (as described in Figure 3a), the suppressive capacity of the sarcoidosis monocytes was also restored to levels observed for monocytes from healthy controls (Figure 6c).

4. Table 2 states that Fig 2A has 7 sarcoid subjects and no HC. But it seems that related results text section reports 12 HC used for the results in Fig 2A and the legend reports 15 HC. Please clarify. Also, the authors created a nice format (table 2) to present the different sample sizes for SOME of the figures, but not ALL of the figures. Why is this? Perhaps making it consistent would make it A LOT easier to follow

(either put all the sample sizes for all figures in the table or get rid of the table and put them in the text, or the figure legends?)

Apologies, yes agree. Fig 2A's reference in Table 2 was a typo – this should state healthy control. We have now kept the table simple and shown demographics for figures where cells were derived from sarcoidosis patient and compared to healthy controls only. For the rest, we have placed the sample sizes in the legend.

5. On pg 10 of results, the authors state that "only CD14+ cells (monocytes) in the co-culture expressed IL-10, implicating these cells as the sole source of IL-10 in the supernatant". But didn't the experimental design consist of sorting out CD14+ monocytes from 36 controls and culturing them with NKT clones? So are you saying that the NKT clone was not the source of IL-10? If this is not correct, please considering clarifying the sentence.

Yes – we sorted out CD14+ cells and cultured with iNKT clones, and when we examined IL-10 expression by FACS in these cells, only CD14+ cells expressed IL-10. This means that iNKT clones is not the source of IL-10.

6. No mention in the statistical section of the manuscript about what type of correlation plot was used.

Have done this now, and also for the new Figures 3B-C.

7. Is reference #35 correctly cited in the text related to NKT cell clones?

Apologies, this should be ref 36, but is now ref 37 due to addition of a reference by the other reviewer.

8. Figure legend 3B. This is not technically a correlation

Thank you, agree. In fact figure 3B and 3C are now modified to remove the arbitrary division to low and high activity. We have amended legend accordingly.

Second Editorial Decision – 17 March 2014

Dear Dr. Ho,

Peer review correspondence

European Journal of Immunology

It is a pleasure to provisionally accept your manuscript entitled "Impaired monocytic IL-10 production in sarcoidosis and potential link to abnormalities in iNKT cells" for publication in the European Journal of Immunology. For final acceptance, please follow the instructions below and return the requested items as soon as possible as we cannot process your manuscript further until all items listed below are dealt with.

Please note that EJI articles are now published online a few days after final acceptance (see Accepted Articles: http://onlinelibrary.wiley.com/journal/10.1002/(ISSN)1521-4141/accepted). The files used for the Accepted Articles are the final files and information supplied by you in Manuscript Central. You should therefore check that all the information (including author names) is correct as changes will NOT be permitted until the proofs stage.

We look forward to hearing from you and thank you for submitting your manuscript to the European Journal of Immunology.

Yours sincerely,
Laura Soto Vazquez

on behalf of
Prof. Iain McInnes

Editorial Office
European Journal of Immunology
e-mail: ejied@wiley.com
www.eji-journal.eu
